# Predicting in-hospital mortality in patients with alcoholic cirrhosis complicated by severe acute kidney injury: development and validation of an explainable machine learning model

**DOI:** 10.3389/fmed.2025.1570928

**Published:** 2025-05-08

**Authors:** Meina Sun, Shihui Liu, Jie Min, Lei Zhong, Jinyu Zhang, Zhian Du

**Affiliations:** ^1^Department of Intensive Care Unit, First Affiliated Hospital of Jinzhou Medical University, Jinzhou, China; ^2^Department of Intensive Care Unit, Huzhou Central Hospital, Affiliated Central Hospital of Huzhou University, Huzhou, China; ^3^Huzhou Central Hospital, Fifth School of Clinical Medicine of Zhejiang Chinese Medical University, Huzhou, China; ^4^Department of General Surgery, Huzhou Central Hospital, Affiliated Central Hospital of Huzhou University, Huzhou, China

**Keywords:** alcoholic cirrhosis, acute kidney Injury, in-hospital mortality, machine learning, predictive model

## Abstract

**Background:**

At present, there are no specialized models for predicting mortality risk in patients with alcoholic cirrhosis complicated by severe acute kidney injury (AKI) in the ICU. This study aims to develop and validate machine learning models to predict the mortality risk of this population during hospitalization.

**Methods:**

A retrospective analysis was conducted on 856 adult patients with alcoholic cirrhosis complicated by severe AKI, utilizing data from the MIMIC-IV database. Within the dataset, 627 patients from the period 2008–2016 were designated as the training cohort, whereas 229 patients from 2017 to 2019 comprised the temporal external validation cohort. Feature selection was conducted utilizing LASSO regression, which was subsequently followed by the development of eight distinct machine learning models. The performance of these models in the temporal external validation cohort was rigorously assessed using the area under the receiver operating characteristic curve (AUROC) to determine the optimal model. The model was interpreted using the SHAP method, and nomograms were subsequently constructed. A comprehensive evaluation was performed from the perspectives of discrimination (assessed via AUROC and AUPRC), calibration (using calibration curves), and clinical utility (evaluated through DCA curves).

**Results:**

LASSO regression identified nine key features: total bilirubin, acute respiratory failure, vasopressin, septic shock, oliguria, AKI stage, lactate, fresh frozen plasma transfusion, and norepinephrine. In the temporal external validation cohort, the Lasso-LR model achieved the highest AUROC value of 0.809, establishing it as the optimal model. We developed both a static nomogram and a web-based dynamic nomogram (https://zhangjingyu123456.shinyapps.io/dynnomapp/) for visualization purposes. In the nomogram model, the AUROC for the training cohort and temporal external validation cohort were 0.836 (95% CI: 0.802-0.870) and 0.809 (95% CI: 0.754–0.865), respectively. The calibration slope and Brier score for the training cohort were 1.000 and 0.146, respectively; for the temporal external validation cohort, these values were 0.808 and 0.177, respectively. The DCA curves indicate that the model has certain clinical application value.

**Conclusion:**

The Lasso-LR model exhibits robust predictive capability for in-hospital mortality among patients with alcoholic cirrhosis complicated by AKI, offering valuable prognostic insights and individualized treatment decision support for healthcare professionals.

## Graphical Abstract

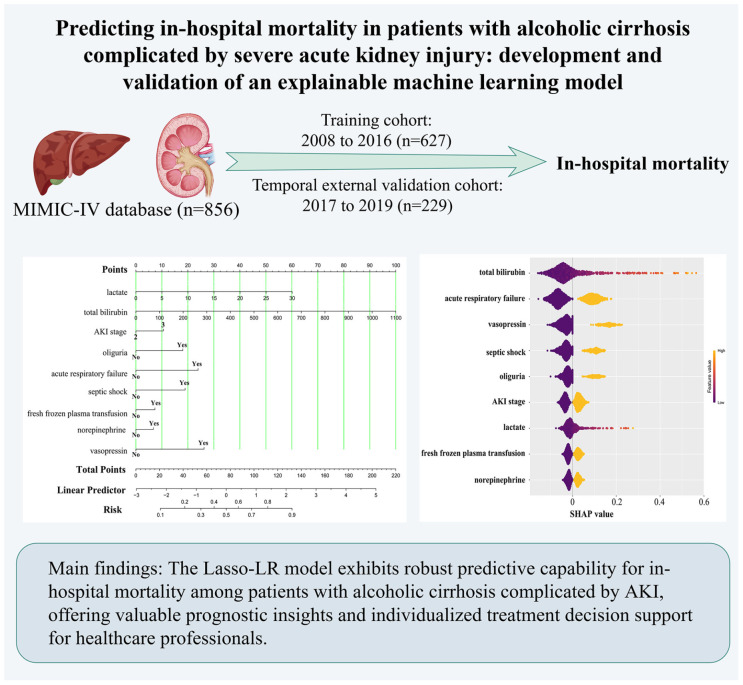

## Introduction

Alcoholic cirrhosis represents a significant hepatic pathology, with global mortality rates attributable to its complications on an upward trajectory (1). The escalation in alcohol consumption is a principal factor contributing to the increasing prevalence of cirrhosis, especially among middle-aged and older male populations (2). In the United States, it is estimated that 23.6 million individuals are affected by alcohol-related cirrhosis, with approximately 2.46 million experiencing decompensated cirrhosis (2). These individuals frequently encounter a range of complications, notably severe acute kidney injury (AKI), which occurs at an incidence rate of 28-65% (3, 4). AKI not only exacerbates the impairment of liver function but also significantly elevates both short-term and long-term mortality rates among patients, thereby intensifying the consumption of healthcare resources and contributing to the economic burden (3, 5, 6). Studies have shown that hospitalized patients with alcoholic cirrhosis who concurrently develop AKI may experience an increase in mortality risk of up to 80% (7). Consequently, the identification and prediction of in-hospital mortality risk in these patients are of paramount clinical importance for optimizing management strategies and enhancing survival outcomes.

Traditional clinical assessment instruments, including the Model for End-Stage Liver Disease (MELD) score and the Child-Turcotte-Pugh (CTP) score, provide a partial reflection of patients’ conditions but frequently fall short in comprehensively addressing the complexities associated with multiple complications (8–10). This limitation is especially evident in patients with alcoholic cirrhosis complicated by severe AKI, where conventional scoring systems may inadequately predict individual mortality risk. Moreover, current prognostic models predominantly utilize statistical methodologies to assess known risk factors for predicting patient mortality. Nonetheless, these models exhibit limitations in identifying intricate data patterns and in their applicability across diverse populations and clinical settings. Presently, there is an absence of specialized prognostic models that effectively encapsulate the complexity inherent in patients with alcoholic cirrhosis complicated by AKI (1, 7, 11). In recent years, advancements in machine learning technologies have offered novel opportunities to enhance the accuracy and interpretability of prognostic models. Explainable machine learning models are capable of processing extensive clinical datasets, identifying potential risk factors, and developing a more adaptable and dynamic prognostic assessment framework. This is essential for improving clinical outcomes and informing personalized treatment strategies (12).

The primary objective of this study is to develop and validate an explainable machine learning model for predicting the risk of in-hospital mortality in patients with alcoholic cirrhosis complicated by severe AKI. Clinical data will be utilized from the large-scale Medical Information Mart for Intensive Care IV (MIMIC-IV 2.0) database, and various machine learning algorithms will be employed to construct prognostic models with clinical applicability. This research aims to provide new perspectives and tools for clinical practice, enhancing the management and prognosis of patients with alcoholic cirrhosis complicated by severe AKI, ultimately improving patient survival rates.

## Materials and methods

### Data source

This study employed the MIMIC-IV database (13), which was developed by the Laboratory for Computational Physiology at the Massachusetts Institute of Technology (MIT). The MIMIC-IV database encompasses data from 76,943 ICU admissions at Beth Israel Deaconess Medical Center (BIDMC) in Boston, Massachusetts, covering the period from 2008 to 2019. The database includes comprehensive information such as demographics, vital signs, laboratory test results, and diagnostic codes, which are aligned with both the International Classification of Diseases, ninth revision (ICD-9), and tenth revision (ICD-10). The utilization of the database has received approval from the Institutional Review Boards (IRBs) of BIDMC and MIT. In accordance with pertinent regulations, all identifiable personal information has been de-identified to ensure patient privacy, obviating the requirement for individual patient consent. Furthermore, all members of the research team have successfully completed compliance training and are certified by the Collaborative Institutional Training Initiative (CITI) program, thereby adhering to established ethical guidelines for human research. This research constitutes a retrospective clinical investigation, wherein model development and validation were performed in compliance with the Transparent Reporting of a Multivariable Prediction Model for Individual Prognosis or Diagnosis (TRIPOD) statement (14).

### Study population

**FIGURE 1:**
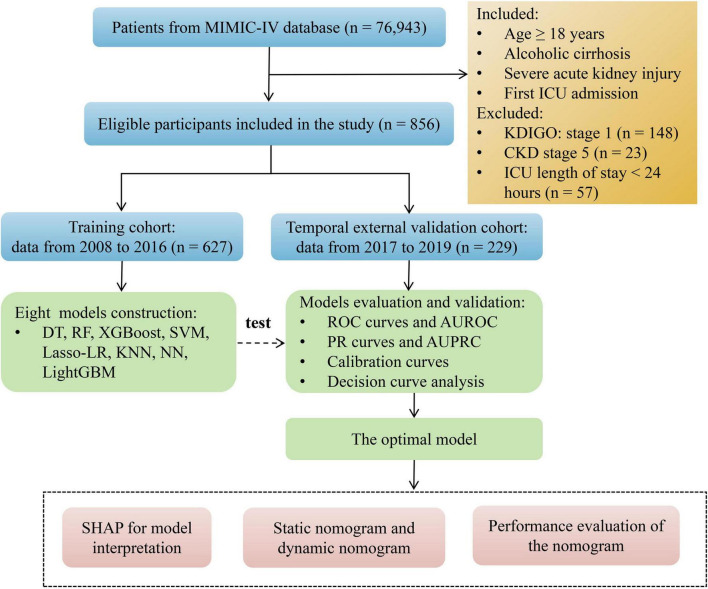
Patient selection process and model construction and validation flowchart. MIMIC-IV, Medical Information Mart for Intensive Care IV; ICU, intensive care unit; KDIGO, Kidney Disease Improving Global Outcomes; CKD, chronic kidney disease; DT, decision tree; RF, random forest; XGBoost, extreme gradient boosting; SVM, support vector machine; Lasso-LR, least absolute shrinkage and selection operator-logistic regression; KNN, k-nearest neighbor; NN, neural network; LightGBM, light gradient boosting machine; ROC, receiver operating characteristic; AUROC, area under the receiver operating characteristic curve; PR, precision-recall; AUPRC, area under the precision-recall curve; SHAP, SHapley Additive exPlanations.

The study encompassed 856 patients diagnosed with alcoholic cirrhosis complicated by AKI from the MIMIC-IV database. The inclusion criteria were as follows: (1) age ≥ 18 years, (2) diagnosis of alcoholic cirrhosis, (3) diagnosis of AKI, and (4) patients with multiple ICU readmissions, with only the first ICU admission considered. The exclusion criteria included: (1) patients with Kidney Disease Improving Global Outcomes (KDIGO) stage 1 AKI, (2) patients with chronic kidney disease (CKD) stage 5, and (3) patients with an ICU length of stay of less than 24 h. Patients treated between 2008 and 2016 (*n* = 627) were utilized to establish the training cohort, whereas those treated from 2017 to 2019 (*n* = 229) constituted the temporal external validation cohort for evaluating the model’s performance. Figure 1 offers a comprehensive depiction of the patient selection process, as well as the flowchart for model construction and validation.

### Definitions and clinical outcomes

AKI was diagnosed within 1 week of hospital admission in accordance with the KDIGO criteria (15). Baseline creatinine values were obtained from the MIMIC-IV database. Severe AKI was defined as stage 2 or 3 AKI (16). Oliguria was defined by a daily urine output of less than 400 mL (17). The primary outcome was in-hospital mortality. In-hospital mortality was defined as all-cause death occurring between admission and discharge, as verified by the definitive discharge status in the hospital’s electronic medical records system.

### Clinical data extraction

In this study, PostgreSQL software (version 14.5) was employed to extract and process data from the MIMIC-IV database. For variables with a missing rate of less than 25%, multiple imputation was conducted using the “mice” package in R, utilizing the predictive mean matching (PMM) method. This process generated five complete datasets and involved 50 iterations. Variables with a missing rate exceeding 25% were excluded from the analysis. The results indicated an absence of missing values among the binary variables. Among the continuous variables, lactate exhibited a missing rate of 24.88%, while the remaining variables demonstrated missing rates below 20%, with the majority showing a missing rate of less than 5%. The resultant dataset included a comprehensive array of information, such as age, sex, Acute Physiology and Chronic Health Evaluation II (APACHE II) score, AKI stage, in-hospital mortality, vital signs, laboratory test results, comorbidities/complications, and therapeutic interventions. Clinical data (vital signs) and laboratory results were extracted from the first recorded measurements obtained during the initial patient assessment in the ICU.

Specifically, the vital signs assessed included heart rate, mean arterial pressure, and respiratory rate. The laboratory test parameters measured encompassed partial pressure of arterial oxygen (PaO2), lactate, anion gap, bicarbonate, blood glucose, white blood cell count (WBC), red blood cell count (RBC), platelet count, red cell distribution width (RDW), mean corpuscular volume (MCV), alanine aminotransferase (ALT), aspartate aminotransferase (AST), total bilirubin, international normalized ratio (INR), blood urea nitrogen (BUN), creatinine, and a range of electrolytes such as sodium, chloride, potassium, total calcium, magnesium, and phosphate.

The study also examined a range of comorbidities and complications, including obesity, diabetes, hypertension, atherosclerotic heart disease, acute myocardial infarction (AMI), atrial fibrillation, congestive heart failure (CHF), chronic obstructive pulmonary disease (COPD), asthma, acute respiratory failure (ARF), cerebral infarction, delirium, depression, CKD, oliguria, sepsis, septic shock, hepatic encephalopathy (HE), and malignant tumors. Therapeutic interventions encompassed the administration of blood products such as RBC transfusion, fresh frozen plasma transfusion, and platelet transfusion, as well as the use of norepinephrine, vasopressin, mechanical ventilation, and renal replacement therapy (RRT).

### Statistical analysis

The quantitative data analysis was performed according to the distributional characteristics of the data. Normally distributed data were expressed as mean ± standard deviation ($\bar{\text{x}}\pm$ s) and analyzed using independent sample *t*-tests. Non-normally distributed data were expressed as median (interquartile range) [M (QL, QU)] and analyzed using the Mann-Whitney *U*-test. Categorical variables were presented as frequencies and percentages, with group comparisons conducted using the chi-square (χ^2^) test or Fisher’s exact test.

To mitigate the risk of overfitting, this study employed least absolute shrinkage and selection operator (LASSO) regression combined with 10-fold cross-validation for feature selection, effectively addressing the variable selection issue. Additionally, a systematic approach combining grid search with 5-fold cross-validation was utilized for hyperparameter tuning to determine the optimal hyperparameter configuration. Based on these methods, eight machine learning predictive models were developed, including decision tree (DT), random forest (RF), extreme gradient boosting (XGBoost), support vector machine (SVM), Lasso-logistic regression (Lasso-LR), k-nearest neighbor (KNN), neural network (NN), and light gradient boosting machine (LightGBM).

Subsequently, the models were evaluated for their discriminatory ability, accuracy, and clinical applicability in the temporal external validation cohort, leading to the selection of the optimal model. The discriminatory ability was assessed using the receiver operating characteristic (ROC) curve and the area under the ROC curve (AUROC), as well as the precision-recall (PR) curve and the area under the precision-recall curve (AUPRC). Accuracy was evaluated using calibration curves and Brier scores. Clinical utility was assessed through decision curve analysis (DCA). To enhance the interpretability of the optimal model, the SHapley Additive exPlanations (SHAP) method was employed to illustrate how each variable contributes to predicted outcome. Finally, the selected predictive model was visualized as static nomograms and a web-based dynamic nomogram to summarize the predictive performance of the optimal model. Data processing and analysis were conducted using Stata version 14.0 (StataCorp, College Station, TX, United States) and R software version 4.2.3 (R Foundation for Statistical Computing, Vienna, Austria). Relevant R packages included glmnet (LASSO regression for variable selection), mice (multiple imputation for handling missing data), and tidymodels (model construction and validation), among others. Results were considered statistically significant at *p* < 0.05 (two-tailed).

## Results

### Study population characteristics

The MIMIC-IV database encompassed 76,943 adult ICU patients, with 1,542 diagnosed with alcoholic cirrhosis. Of these, 1,084 also presented with AKI. To focus on severe AKI, 148 patients in KDIGO stage 1 were excluded from the analysis. Additionally, 23 patients in CKD stage 5 and 57 patients with an ICU length of stay of less than 24 h were also excluded. Ultimately, the study included 856 patients with alcoholic cirrhosis complicated by severe AKI. The demographic breakdown included 598 males (69.86%), with an average age of 56.80 ± 11.29 years and an APACHE II score of 24.63 ± 6.97. According to KDIGO staging, 382 patients were in stage 2 and 474 in stage 3. The in-hospital mortality for this population was 34.40% (*n* = 295). Patients were sequentially assigned to a training cohort (*n* = 627) and a temporal external validation cohort (*n* = 229). Additionally, patients were categorized into a survival group (*n* = 561) and a non-survivor group (*n* = 295) based on their hospitalization outcomes. Table 1 presents a comparative analysis of the general characteristics across the training and temporal external validation cohorts, as well as between survivors and non-survivors.

**TABLE 1:** The general characteristics of patients with alcoholic cirrhosis complicated by severe acute kidney injury in different groups.

Characteristic	Total (*n* = 856)	Training cohort (*n* = 627)	External validation cohort (*n* = 229)	t/Z/χ ^2^	*P*-value	Survivors (*n* = 561)	Non-survivors (*n* = 295)	t/Z/χ 2	*P*-value
**Demographics**									
Age (years)	56.80 ± 11.29	57.44 ± 10.96	55.04 ± 11.99	2.765	0.006	56.77 ± 11.31	56.85 ± 11.27	–0.095	0.925
Male, n (%)	598 (69.86)	445 (70.97)	153 (66.81)	1.379	0.240	402 (71.66)	196 (66.44)	2.499	0.114
APACHE II score (score)	24.63 ± 6.97	24.69 ± 6.83	24.45 ± 7.35	0.458	0.647	23.36 ± 6.86	27.03 ± 6.54	–7.565	<0.001
**AKI stage, n (%)**									
Stage 2	382 (44.63)	294 (46.89)	88 (38.43)	4.861	0.027	310 (55.26)	72 (24.41)	74.469	<0.001
Stage 3	474 (55.37)	333 (53.11)	141 (61.57)			251 (44.74)	223 (75.59)		
**Vital signs**									
Heart rate (beats/min)	95.14 ± 20.14	94.59 ± 20.50	96.66 ± 19.07	–1.335	0.182	94.12 ± 20.16	97.07 ± 19.99	–2.038	0.042
Mean arterial pressure (mmHg)	80.89 ± 18.22	80.46 ± 18.70	82.07 ± 16.85	–1.145	0.252	81.76 ± 18.27	79.24 ± 18.05	1.925	0.055
Respiratory rate (beats/min)	20.33 ± 6.10	20.13 ± 6.01	20.89 ± 6.32	–1.602	0.109	19.70 ± 5.89	21.54 ± 6.31	–4.250	<0.001
**Laboratory tests**									
PaO2 (mmHg)	78.50 (50.00, 146.50)	88.00 (54.00, 169.00)	60.00 (41.00, 101.00)	6.425	<0.001	82.00 (52.00, 169.00)	73.00 (46.00, 121.00)	2.948	0.003
Lactate (mmol/L)	2.20 (1.60,3.50)	2.10 (1.60,3.50)	2.30 (1.60,3.60)	–0.543	0.587	2.00 (1.50, 3.00)	2.60 (1.80, 4.50)	–6.364	<0.001
Anion gap (mmol/L)	17.36 ± 6.24	17.41 ± 6.63	17.24 ± 5.03	0.358	0.721	16.56 ± 5.94	18.88 ± 6.52	–5.256	<0.001
Bicarbonate (mmol/L)	20.79 ± 5.26	20.99 ± 5.36	20.24 ± 4.94	1.868	0.062	21.36 ± 5.07	19.70 ± 5.44	4.442	<0.001
Blood glucose (mmol/L)	6.72 (5.61, 8.86)	6.72 (5.61, 8.94)	6.83 (5.61, 8.33)	0.172	0.864	6.89 (5.72, 9.28)	6.50 (5.28, 8.28)	3.325	<0.001
WBC ( × 10^9^/L)	10.00 (6.60, 15.05)	9.90 (6.50, 14.60)	11.00 (7.00, 16.30)	–2.278	0.023	9.20 (6.20, 13.70)	11.80 (8.00, 17.40)	–5.346	<0.001
RBC ( × 10^12^/L)	3.02 ± 0.79	3.06 ± 0.79	2.92 ± 0.79	2.160	0.031	3.11 ± 0.80	2.85 ± 0.75	4.504	<0.001
Platelet ( × 10^9^/L)	101.00 (67.00, 155.00)	102.00 (66.00, 154.00)	100.00 (67.00, 156.00)	0.176	0.861	103.00 (71.00, 160.00)	95.00 (60.00, 141.00)	2.648	0.008
RDW (%)	17.48 ± 3.00	17.35 ± 2.90	17.84 ± 3.23	–2.089	0.037	17.14 ± 2.87	18.12 ± 3.14	–4.601	<0.001
MCV (fL)	98.30 ± 9.25	98.30 ± 9.28	98.29 ± 9.17	0.019	0.985	96.99 ± 8.77	100.78 ± 9.64	–5.792	<0.001
ALT (U/L)	36.00 (22.00, 67.00)	36.00 (22.00, 67.00)	33.00 (21.00, 72.00)	0.701	0.483	34.00 (21.00, 72.00)	40.00 (24.00, 63.00)	–1.280	0.201
AST (U/L)	83.00 (45.00, 167.00)	83.00 (45.00, 164.00)	82.00 (49.00, 183.00)	–0.670	0.503	77.00 (42.00, 164.00)	92.00 (55.00, 176.00)	–2.574	0.010
Total bilirubin (umol/L)	72.68 (30.78, 179.55)	66.69 (29.07, 165.87)	90.63 (39.33, 241.11)	–3.729	<0.001	53.01 (25.65, 124.83)	138.51 (53.01, 333.45)	–9.548	<0.001
INR	1.80 (1.50, 2.40)	1.70 (1.40, 2.30)	1.90 (1.50, 2.50)	–2.614	0.009	1.60 (1.40, 2.10)	2.10 (1.70, 2.80)	–9.969	<0.001
BUN (mmol/L)	8.90 (4.98, 15.84)	8.90 (4.98, 16.02)	8.19 (4.98, 15.66)	0.675	0.500	7.48 (4.63, 13.88)	12.46 (6.41, 19.94)	–6.061	<0.001
Creatinine (umol/L)	106.08 (70.72, 203.32)	106.08 (70.72, 203.32)	114.92 (79.56, 185.64)	–0.605	0.545	97.24 (70.72, 167.96)	141.44 (88.40, 238.68)	–6.222	<0.001
Sodium (mmol/L)	135.64 ± 7.50	135.62 ± 7.27	135.69 ± 8.12	–0.115	0.908	136.11 ± 6.91	134.74 ± 8.46	2.537	0.011
Chlorine (mmol/L)	100.51 ± 9.10	101.10 ± 8.80	98.89 ± 9.72	3.163	0.002	101.28 ± 8.65	99.04 ± 9.75	3.437	<0.001
Potassium (mmol/L)	4.29 ± 0.95	4.26 ± 0.95	4.37 ± 0.93	–1.526	0.127	4.27 ± 0.89	4.32 ± 1.04	–0.809	0.419
Total calcium (mmol/L)	2.06 ± 0.28	2.06 ± 0.29	2.07 ± 0.23	–0.121	0.904	2.05 ± 0.28	2.08 ± 0.27	–1.448	0.148
Magnesium (mmol/L)	0.80 ± 0.20	0.80 ± 0.20	0.79 ± 0.17	1.192	0.234	0.78 ± 0.18	0.83 ± 0.21	–3.883	<0.001
Phosphate (mmol/L)	1.33 ± 0.58	1.32 ± 0.58	1.36 ± 0.59	–0.777	0.437	1.28 ± 0.52	1.43 ± 0.67	–3.792	<0.001
**Comorbidities/complications, n (%)**									
Obesity	280 (32.74)	204 (35.43)	76 (27.10)	0.032	0.857	172 (29.86)	108 (38.57)	3.110	0.078
Diabetes	170 (19.90)	132 (19.20)	38 (22.40)	2.095	0.148	116 (16.93)	54 (31.76)	0.684	0.408
Hypertension	291 (34.03)	214 (34.11)	77 (26.50)	0.019	0.890	203 (35.91)	88 (29.76)	3.480	0.062
Atherosclerotic heart disease	43 (5.02)	20 (3.19)	23 (53.49)	16.515	<0.001	31 (3.82)	12 (27.91)	0.862	0.353
AMI	37 (4.32)	23 (3.67)	14 (37.84)	2.425	0.119	17 (3.03)	20 (7.27)	6.572	0.010
Atrial fibrillation	145 (17.00)	112 (17.80)	33 (22.83)	1.421	0.233	88 (12.39)	57 (39.31)	1.816	0.178
CHF	124 (14.46)	99 (15.81)	25 (10.87)	3.215	0.073	81 (12.60)	43 (34.68)	0.003	0.957
COPD	101 (11.82)	74 (9.80)	27 (26.73)	0.000	0.996	72 (9.57)	29 (28.71)	1.676	0.195
Asthma	59 (6.92)	50 (6.90)	9 (15.25)	4.275	0.039	44 (8.48)	15 (5.08)	2.292	0.130
ARF	371 (43.36)	251 (41.30)	120 (32.26)	10.452	<0.001	187 (38.56)	184 (62.07)	66.393	<0.001
Cerebral infarction	46 (5.42)	37 (4.60)	9 (19.57)	1.281	0.258	35 (6.54)	11 (4.05)	2.395	0.122
Delirium	70 (8.17)	43 (5.50)	27 (38.64)	5.435	0.020	53 (10.49)	17 (5.75)	3.496	0.062
Depression	170 (19.84)	120 (17.39)	50 (29.41)	0.766	0.382	116 (16.92)	54 (31.76)	0.684	0.408
CKD	99 (11.56)	76 (10.10)	23 (23.19)	0.708	0.400	61 (8.06)	38 (38.38)	0.762	0.383
Oliguria	181 (21.10)	117 (18.60)	64 (27.90)	8.677	<0.001	73 (12.56)	108 (31.67)	64.568	<0.001
Sepsis	702 (82.03)	515 (82.14)	187 (81.66)	0.026	0.872	443 (79.36)	259 (87.88)	10.218	0.001
Septic shock	226 (26.43)	147 (23.41)	79 (34.88)	10.546	<0.001	90 (14.29)	136 (60.18)	89.899	<0.001
HE	161 (18.92)	161 (22.30)	0 (0.00)	72.424	<0.001	97 (13.96)	64 (40.00)	2.456	0.117
Malignant tumor	124 (14.46)	98 (13.40)	26 (21.00)	2.476	0.116	78 (10.69)	46 (15.59)	0.445	0.504
**Treatments, n (%)**									
Blood transfusion	587 (68.49)	418 (68.20)	169 (73.75)	3.960	0.047	366 (65.61)	221 (74.74)	8.397	0.004
RBC transfusion	498 (58.19)	356 (57.20)	142 (62.30)	1.886	0.170	318 (56.70)	180 (61.02)	1.492	0.222
Fresh frozen plasma transfusion	367 (42.92)	267 (42.60)	100 (43.60)	0.081	0.777	188 (34.24)	179 (60.68)	58.257	<0.001
Platelet transfusion	295 (34.40)	204 (32.60)	91 (41.00)	3.852	0.050	183 (32.61)	112 (37.97)	2.446	0.118
Norepinephrine	414 (48.29)	268 (43.10)	146 (63.10)	29.655	<0.001	206 (46.62)	208 (50.00)	88.383	<0.001
Vasopressin	193 (22.63)	111 (18.00)	82 (35.80)	31.483	<0.001	63 (9.52)	130 (67.42)	119.382	<0.001
Mechanical ventilation	629 (73.51)	448 (71.51)	181 (80.00)	4.956	0.026	383 (68.62)	246 (83.79)	22.679	<0.001
RRT	178 (20.83)	107 (17.10)	71 (29.50)	19.787	<0.001	78 (11.52)	100 (56.18)	46.928	<0.001
**Outcome**									
In-hospital mortality, n (%)	295 (34.40)	204 (32.54)	91 (39.74)	3.852	0.050				

APACHE II, Acute Physiology and Chronic Health Evaluation II; AKI, acute kidney injury; PaO_2_, partial pressure of arterial oxygen; WBC, white blood cell; RBC, red blood cell; RDW, red cell distribution width; MCV, mean corpuscular volume; ALT, alanine transaminase; AST, aspartate transaminase; INR, international normalized ratio; BUN, blood urea nitrogen; AMI, acute myocardial infarction; CHF, congestive heart failure; COPD, chronic obstructive pulmonary disease; ARF, acute respiratory failure; CKD, chronic kidney disease; HE, hepatic encephalopathy; RRT, renal replacement therapy.

### Feature selection for predicting in-hospital mortality

**FIGURE 2:**
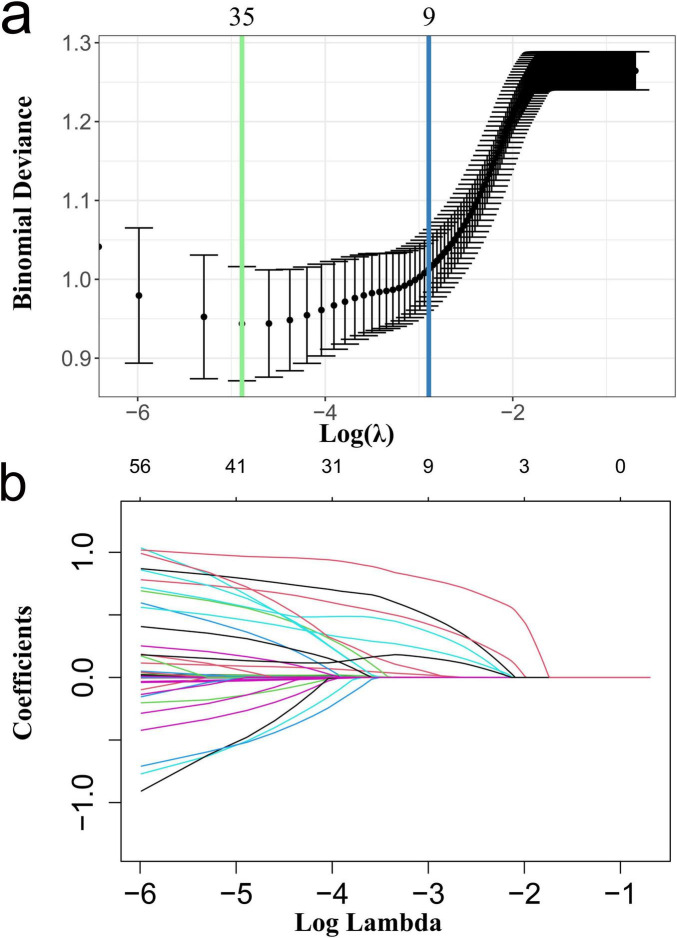
Clinical features were selected based on LASSO regression with cross-validation. **(a)** Selection of optimal parameter lambda (cross-validation); **(b)** Dynamic process diagram of feature selection using LASSO regression. LASSO, least absolute shrinkage and selection operator.

In this study, in-hospital mortality among patients with alcoholic cirrhosis complicated by AKI was designated as the dependent variable. A total of 56 clinical features from the training cohort were utilized as independent variables. Lasso regression analysis was employed to identify factors significantly associated with in-hospital mortality. To prevent model overfitting, cross-validation was conducted, determining the optimal λ (lambda) value at 0.055 (refer to Figure 2). Consequently, nine key features were identified: lactate, total bilirubin, AKI classification, acute respiratory failure, septic shock, oliguria, vasopressin, norepinephrine, and fresh frozen plasma transfusion.

### Construction and evaluation of mortality prediction models

In this study, nine key clinical features were utilized across various machine learning models, including DT, RF, XGBoost, SVM, Lasso-LR, KNN, NN, and LightGBM. To optimize model performance, five-fold cross-validation was employed for parameter tuning, and multiple iterations of model training were conducted to identify the most effective predictive model. The performance metrics of the eight machine learning models, evaluated on both the training cohort and the temporal external validation cohort, are presented in Table 2 and Supplementary Figures 1–4.

**TABLE 2:** Performance assessment of eight algorithmic models in both training and temporal external validation cohorts.

Models	AUROC (95%CI)	Sensitivity (%)	Specificity (%)	accuracy	Kappa
**Training cohort**					
Decision tree	0.843 (0.807–0.878)	0.686	0.903	0.833	0.607
Random forest	0.870 (0.842–0.899)	0.789	0.797	0.794	0.556
XGBoost	0.837 (0.804–0.870)	0.809	0.735	0.759	0.498
Support vector machine	0.836 (0.803–0.870)	0.750	0.780	0.770	0.503
Lasso-LR	0.836 (0.802–0.870)	0.750	0.780	0.770	0.503
K-nearest neighbor	0.907 (0.884–0.929)	0.833	0.813	0.820	0.612
Neural network	0.830 (0.795–0.864)	0.696	0.837	0.791	0.528
LightGBM	0.856 (0.824–0.887)	0.779	0.790	0.786	0.539
**Temporal external validation cohort**					
Decision tree	0.723 (0.656–0.791)	0.725	0.674	0.694	0.385
Random forest	0.801 (0.744–0.858)	0.846	0.580	0.686	0.393
XGBoost	0.800 (0.743–0.856)	0.868	0.551	0.677	0.383
Support vector machine	0.800 (0.744–0.857)	0.824	0.565	0.668	0.360
Lasso-LR	0.809 (0.754–0.865)	0.857	0.609	0.707	0.433
K-nearest neighbor	0.772 (0.709–0.834)	0.791	0.580	0.664	0.346
Neural network	0.800 (0.743-0.857)	0.791	0.652	0.707	0.421
LightGBM	0.808 (0.752–0.864)	0.857	0.580	0.690	0.403

AUROC, area under the receiver operating characteristic curve; CI, confidence interval; XGBoost, extreme gradient boosting; Lasso-LR, least absolute shrinkage and selection operator-logistic regression; LightGBM, light gradient boosting machine.

In the training cohort, the AUROC for all models surpassed 0.830, indicating robust predictive performance. In the temporal external validation cohort, the Lasso-LR model achieved the highest AUROC value of 0.809, followed sequentially by the LightGBM, RF, XGBoost, SVM, and NN models. Conversely, the DT model exhibited the lowest AUROC at 0.723 (refer to Supplementary Figure 1). In the PR curve analysis, we found that the SVM and XGBoost models exhibited higher AUPRC values of 0.730 and 0.727, respectively, indicating their strong capability in identifying positive cases across different thresholds. Other models such as RF, NN, Lasso-LR, and LightGBM also showed decent AUPRC values of 0.718, 0.718, 0.714, and 0.711, respectively, demonstrating a good balance between precision and recall (refer to Supplementary Figure 2). Furthermore, calibration plots for the eight models in the temporal external validation cohort (refer to Supplementary Figure 3) indicated satisfactory calibration performance, with the Lasso-LR model demonstrating an excellent fit, as evidenced by a Brier score of 0.177. Moreover, DCA curves demonstrated that all eight models exhibited clinical utility (refer to Supplementary Figure 4), thereby substantiating their potential for future clinical decision-making. Notably, the Lasso-LR model exhibited superior performance in the DCA curve, with its net benefit exceeding that of the other models across a wide spectrum of thresholds. This finding implies that the Lasso-LR model may be the most advantageous option for practical clinical applications.

### SHAP for model interpretation

**FIGURE 3:**
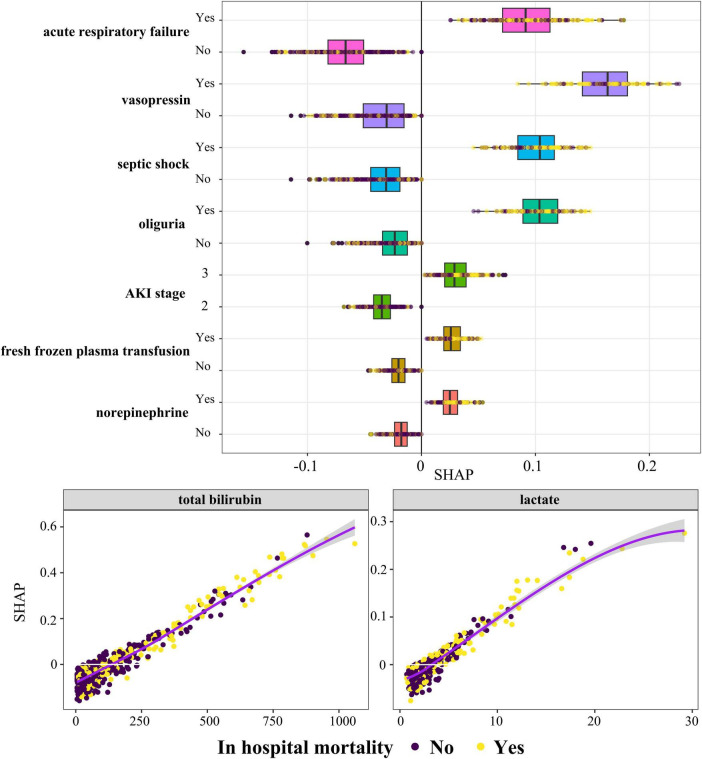
The predictive impact of nine clinical features on in-hospital mortality risk. SHAP values indicate the contribution direction and strength of each feature to the predictive model. Positive values signify an increased risk of mortality, while negative values indicate a reduced risk. Color coding: 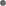 Black represents surviving cases, and 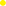 Yellow represents in-hospital mortality cases. AKI, acute kidney injury; SHAP, SHapley Additive exPlanations.

To elucidate the role of the selected variables, we utilized the SHAP algorithm to quantify the contribution of each feature to the prediction of in-hospital mortality within the Lasso-LR model. By plotting the SHAP values for each sample, we were able to visually assess the impact of each feature on the prediction outcome. Supplementary Figure 5 presents a beeswarm plot that ranks the importance of the features in descending order, highlighting the following variables as most significant: total bilirubin, acute respiratory failure, vasopressin, septic shock, oliguria, AKI classification, lactate, fresh frozen plasma transfusion, and norepinephrine. Figure 3 provides a detailed depiction of the specific impact of these features on in-hospital mortality rates. It is noteworthy that all nine features exhibited a positive correlation with higher SHAP values, suggesting that an increase in these characteristics is associated with an elevated risk of in-hospital mortality.

### Nomogram for predicting in-hospital mortality

**FIGURE 4:**
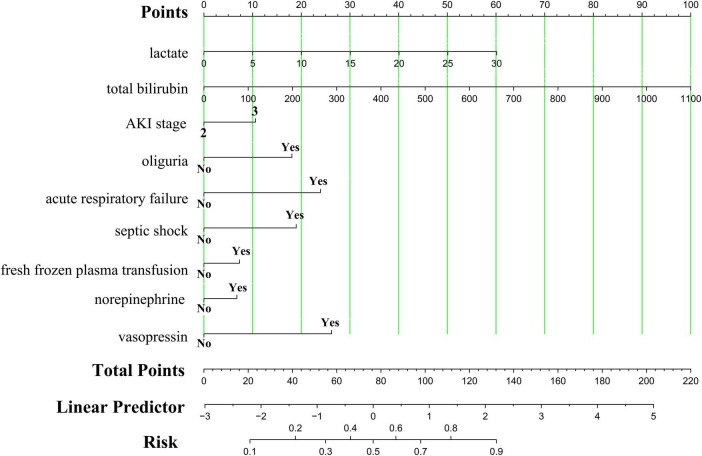
Nomogram for predicting in-hospital mortality in patients with alcoholic cirrhosis complicated by severe acute kidney injury. AKI, acute kidney injury.

In this study, we employed a nomogram tool to convert nine key predictive factors derived from the Lasso-LR model into a mortality risk assessment for patients with alcoholic cirrhosis complicated by severe AKI. The nomogram assigns specific scores to each predictive variable, aggregates these scores to produce a total score, and subsequently translates this total score into a probability of mortality. Utilizing R software, we streamlined the model into a static nomogram for ease of interpretation, as depicted in Figure 4. Clinicians can evaluate the mortality risk in patients through the computation of a composite score derived from the nine parameters. Furthermore, we have developed a web-based dynamic nomogram, accessible at https://zhangjingyu123456.shinyapps.io/dynnomapp/, to provide healthcare professionals with convenient access via smartphones and computers.

For instance, when a patient with alcoholic cirrhosis complicated by severe AKI meets the following criteria (blood lactate concentration of 30 mmol/L, total bilirubin concentration of 50 μmol/L, reaches AKI stage 3, accompanied by oliguria and septic shock, without acute respiratory failure, receiving norepinephrine treatment, and without fresh frozen plasma transfusion or vasopressin use), the nomogram model indicates that the in-hospital mortality probability for this patient is 84.30%, as detailed in Supplementary Figure 6.

### Performance evaluation of the nomogram

**FIGURE 5:**
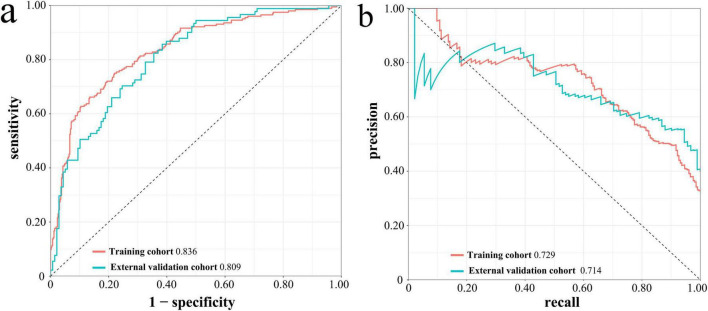
ROC curves **(a)** and PR curves **(b)** of Lasso-LR model in the training cohort and the temporal external validation cohort. ROC, receiver operating characteristic; PR, precision-recall; Lasso-LR, lasso-logistic regression.

In the Lasso-LR model, the AUROC for the training cohort and the temporal external validation cohort were 0.836 (95% CI: 0.802-0.870) and 0.809 (95% CI: 0.754-0.865), respectively, as illustrated in Figure 5a. These results indicate that the model exhibits robust discriminative performance in predicting in-hospital mortality among patients. Furthermore, the PR curves, depicted in Figure 5b, corroborates the model’s strong discriminative capability.

**FIGURE 6:**
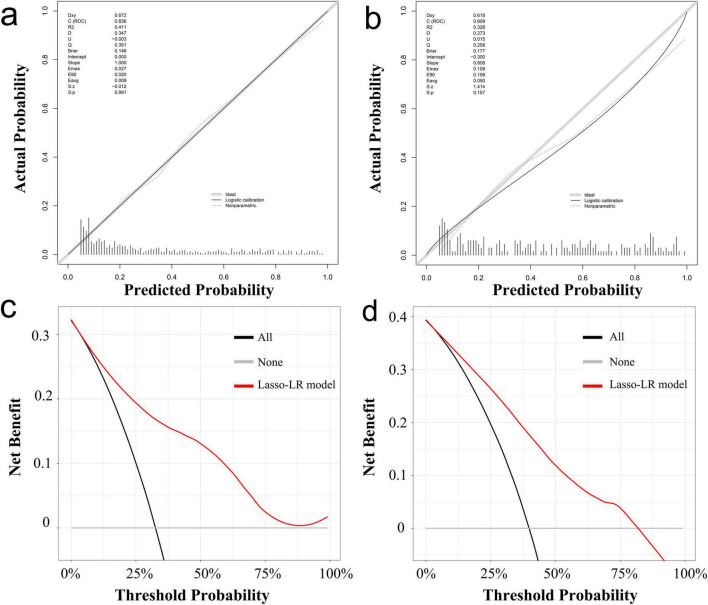
Calibration curves **(a,b)** and decision curve analysis **(c,d)** of Lasso-LR model in the training cohort and the temporal external validation cohort. Lasso-LR, lasso-logistic regression.

The calibration curves for the training cohort and the temporal external validation cohort are shown in Figures 6a,b, respectively. The calibration slope for the training cohort was 1.000, accompanied by a Brier score of 0.146, whereas the calibration slope for the temporal external validation cohort was 0.808, with a Brier score of 0.177. These results suggest a high degree of concordance between the predicted and observed outcomes. Additionally, the close alignment of the calibration curves with the ideal curve further substantiates the model’s high accuracy across the entire dataset.

The DCA curves indicate that, over a broad spectrum of threshold probabilities, the model’s predictions for in-hospital mortality outperform both the “treat all” and “treat none” strategies. This superiority is evident in both the training cohort and the temporal external validation cohort, where the model exhibits a favorable net benefit, thereby underscoring its potential clinical utility, as illustrated in Figures 6c,d.

## Discussion

This study developed and validated various machine learning models to predict the risk of in-hospital mortality in patients with alcoholic cirrhosis complicated by severe AKI. By analyzing clinical data from the MIMIC-IV database, the Lasso-LR model was identified as outperforming other models in the temporal external validation cohort, achieving a high AUROC and a low Brier score, demonstrating excellent predictive performance. This finding not only provides a novel tool for assessing the mortality risk in patients with alcoholic cirrhosis complicated by severe AKI but also offers a basis for clinical decision support in practical applications.

The findings of our study suggest that the nine identified key features possess substantial clinical significance in forecasting in-hospital mortality. Elevated total bilirubin levels generally indicate compromised liver function, especially in patients with alcoholic cirrhosis. At this juncture, the liver’s capacity to metabolize and excrete bilirubin diminishes, resulting in heightened serum bilirubin concentrations. This condition is frequently linked to the onset of AKI, and in patients with cirrhosis, direct bilirubin levels are regarded as a critical predictor of AKI development (18). In the context of AKI, elevated bilirubin levels may indicate concurrent hepatic and renal damage, a condition frequently observed in patients with alcoholic cirrhosis (19). A recent study employed nomograms to predict in-hospital mortality among patients with cirrhosis complicated by AKI, revealing that total bilirubin serves as a prognostic indicator for mortality within this cohort (7). Furthermore, animal studies have demonstrated that hyperbilirubinemia can induce pro-apoptotic effects and exacerbate renal ischemia-reperfusion injury (20).

In patients with cirrhosis and AKI, oliguria serves as a significant risk factor for mortality. As a clinical manifestation of AKI, oliguria indicates the severity of tubular damage and is strongly associated with increased mortality rates (21). This study suggested an association between the stages of AKI and patient mortality, aligning with findings from previous research (11, 22). These results underscore the importance of early recognition and intervention in the management of AKI by clinicians. Moreover, the onset of acute respiratory failure results in a systemic reduction in oxygenation, which exacerbates hepatic injury and triggers a systemic inflammatory response, consequently elevating the risk of mortality. Studies suggest that acute respiratory failure frequently correlates with infections, deterioration of liver function, and dysfunction of other organs, all of which substantially increase the mortality risk in patients with cirrhosis (23). Furthermore, hypoxia can induce metabolic abnormalities, biochemical disturbances, and structural dysfunction in renal tubular epithelial cells. These alterations trigger inflammatory responses and generate reactive oxygen species, thereby exacerbating the pathological changes in the kidneys associated with hypoxic injury (24).

According to the Third International Consensus Definitions for Sepsis and Septic Shock (Sepsis-3), septic shock is delineated as a specific form of sepsis marked by sustained hypotension that necessitates the use of vasopressors to maintain a mean arterial pressure of at least 65 mmHg, despite adequate volume resuscitation, alongside serum lactate levels exceeding 2 mmol/L (25). In individuals with alcoholic cirrhosis, the incidence of septic shock is frequently associated with systemic inflammatory responses that result in compromised microcirculatory perfusion, thereby aggravating renal injury and contributing to multiple organ dysfunction. A study examining patients with cirrhosis in the ICU demonstrated a significant association between septic shock and the onset of AKI, with an incidence rate of AKI reaching up to 61% among these patients (3). Elevated lactate levels are indicative not only of tissue hypoxia and microcirculatory disturbances but are also associated with the diminished capacity of the liver to metabolize lactate in cases of advanced liver cirrhosis (26). Microcirculatory dysfunction is posited to play a pivotal role in the pathophysiology, potentially resulting in hepatic hypoxia and subsequent deterioration of renal function (27). Furthermore, vasoactive agents, including vasopressin and norepinephrine, are frequently administered to critically ill patients to manage hypotension and sustain circulatory volume. Nevertheless, the overuse of these agents can lead to peripheral vasoconstriction, which may further diminish renal blood flow, exacerbate AKI, and elevate mortality risk (28). Empirical studies have demonstrated a correlation between vasopressin administration and a heightened incidence of AKI, particularly among critically ill patients (4). Moreover, the administration of vasopressin can result in electrolyte imbalances, thereby exacerbating the risk of renal dysfunction (29). In individuals with alcoholic cirrhosis, norepinephrine treatment is frequently linked to elevated incidences of liver dysfunction and multiple organ failure, further compounding the risk of an unfavorable prognosis (30). While the infusion of fresh frozen plasma is employed to address coagulopathy, excessive transfusion in some instances may lead to volume overload, negatively impacting cardiac and renal function. Therefore, while the transfusion of fresh frozen plasma is necessary in acute hemorrhage situations, the balance of risks and benefits in some patients may impact inpatient mortality rates.

In summary, the nine clinical features identified through LASSO regression are associated with in-hospital mortality in patients with alcoholic cirrhosis complicated by AKI. The Lasso-LR model demonstrates potential clinical utility, as evidenced by a high concordance between predicted and observed risks in the calibration curve and potential net clinical benefits across decision thresholds in the DCA. Based on the current findings, clinicians may consider incorporating the model’s predictions as a supplementary tool for individualized treatment planning, though its clinical applicability requires further validation in prospective cohorts and external settings.

The findings of this study bear substantial implications for clinical practice. Firstly, this research addresses a relatively underexplored cohort of patients with alcoholic cirrhosis complicated by severe acute kidney injury (AKI), thereby contributing novel insights to the field. Secondly, the study utilized LASSO regression for feature selection and developed various machine learning models, undertaking a thorough evaluation of model performance through the use of ROC curves, PC curves, calibration curves, and DCA curves. Furthermore, the application of SHAP methods to interpret the models significantly enhances their interpretability. Finally, the study successfully developed both static and dynamic nomograms to enhance usability and comprehension for clinicians. This study is subject to certain limitations. First, the research data were exclusively sourced from the MIMIC-IV database, which is derived from a single institution, Beth Israel Deaconess Medical Center. Consequently, potential systematic bias may exist due to institution-specific demographic distributions (e.g., race, age) and clinical protocols (e.g., medication regimens, critical care standards). These factors necessitate cautious interpretation when attempting to generalize the findings to populations with diverse ethnicities, geographic regions, or healthcare systems. Second, while the model was validated using a temporal external cohort, its generalizability in other clinical settings (e.g., primary care hospitals and non-European/American healthcare centers) requires further verification. Future work will focus on prospective validation studies in multicenter clinical environments, utilizing real-world data from diverse populations to assess the model’s practical applicability. Additionally, we plan to refine the algorithm to enhance its stability and adaptability in heterogeneous clinical scenarios.

## Conclusion

The Lasso-LR model exhibits robust predictive capability for in-hospital mortality among patients with alcoholic cirrhosis complicated by AKI, offering valuable prognostic insights and individualized treatment decision support for healthcare professionals. Future research should aim to further investigate the applicability of these features across diverse populations, optimize risk prediction tools, and improve the precision and efficacy of clinical management strategies.

## Data Availability

Publicly available datasets were analyzed in this study. This data can be found at: https://physionet.org/content/mimiciv/2.0/.

## Supplementary material

Supplementary Figure 1 AUROCs of eight machine learning models in the temporal external validation cohort.AUROC, area under the receiver operating characteristic curve; XGBoost, extreme gradient boosting; Lasso-LR, lasso-logistic regression; LightGBM, light gradient boosting machine.

Supplementary Figure 2 AUPRCs of eight machine learning models in the temporal external validation cohort.AUPRC, area under the precision-recall curve; XGBoost, extreme gradient boosting; Lasso-LR, lasso-logistic regression; LightGBM, light gradient boosting machine.

Supplementary Figure 3 Calibration curves of eight machine learning models in the temporal external validation cohort.DT, decision tree; RF, random forest; XGBoost, extreme gradient boosting; SVM, support vector machine; Lasso-LR, least absolute shrinkage and selection operator-logistic regression; KNN, k-nearest neighbor; NN, neural network; LightGBM, light gradient boosting machine.

Supplementary Figure 4 Decision curve analysis of eight machine learning models in the temporal external validation cohort.DT, decision tree; RF, random forest; XGBoost, extreme gradient boosting; SVM, support vector machine; Lasso-LR, least absolute shrinkage and selection operator-logistic regression; KNN, k-nearest neighbor; NN, neural network; LightGBM, light gradient boosting machine.

Supplementary Figure 5 Beeswarm plot of feature importance ranked by SHAP values in the Lasso-LR model.SHAP, SHapley Additive exPlanations; Lasso-LR, lasso-logistic regression; AKI, acute kidney injury.

Supplementary Figure 6 Example case using the web-based dynamic nomogram.
