# Supplementary figures and images for: Predicting in-hospital mortality in patients with alcoholic cirrhosis complicated by severe acute kidney injury: development and validation of an explainable machine learning model
Source: Front Med (Lausanne). 2025 May 8;12:1570928. doi: 10.3389/fmed.2025.1570928 (PMC12095237; doi:10.3389/fmed.2025.1570928)

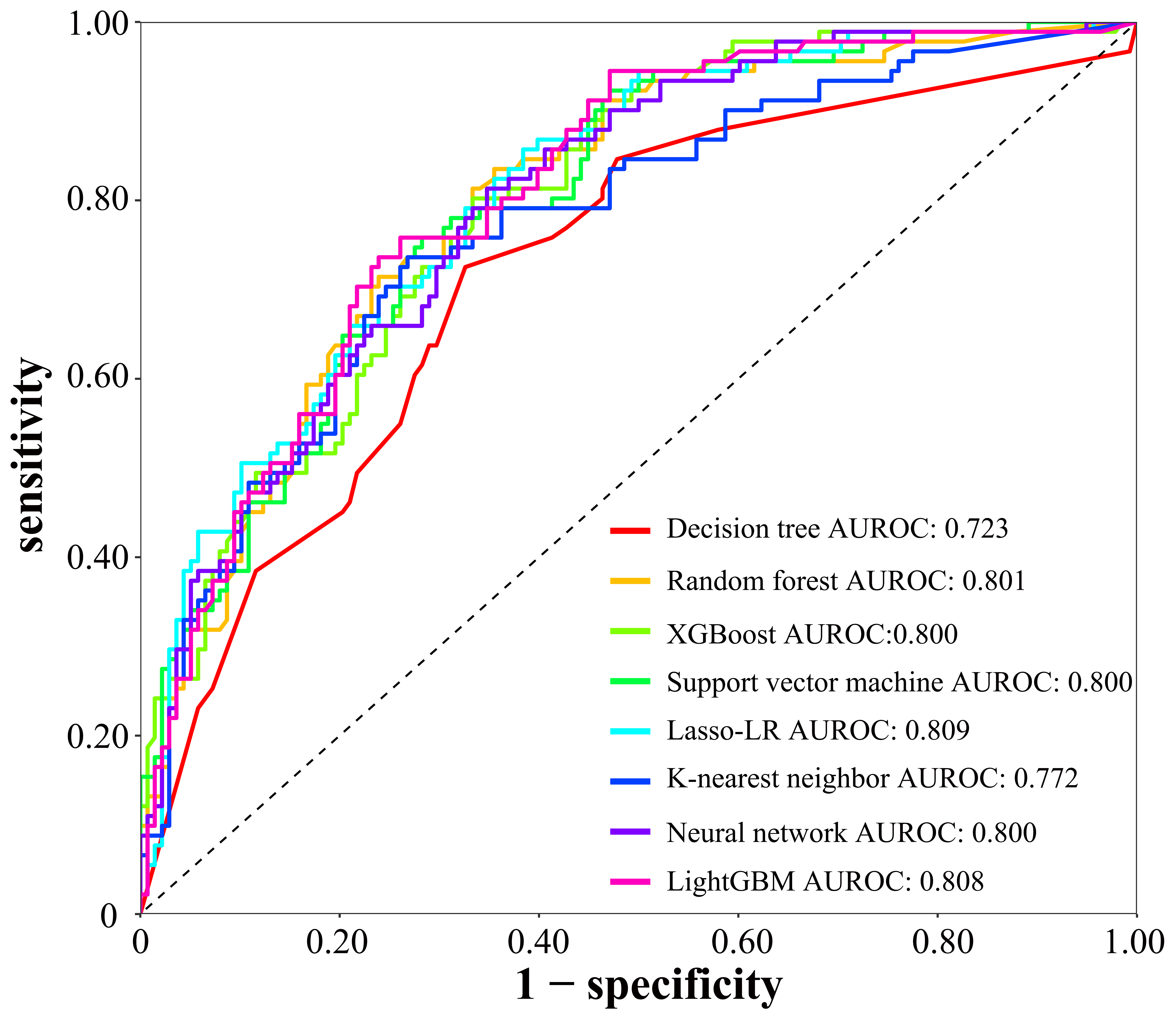

Supplement: Supplementary Figure 1 — AUROCs of eight machine learning models in the temporal external validation cohort.AUROC, area under the receiver operating characteristic curve; XGBoost, extreme gradient boosting; Lasso-LR, lasso-logistic regression; LightGBM, light gradient boosting machine. [file Image_1.tif]

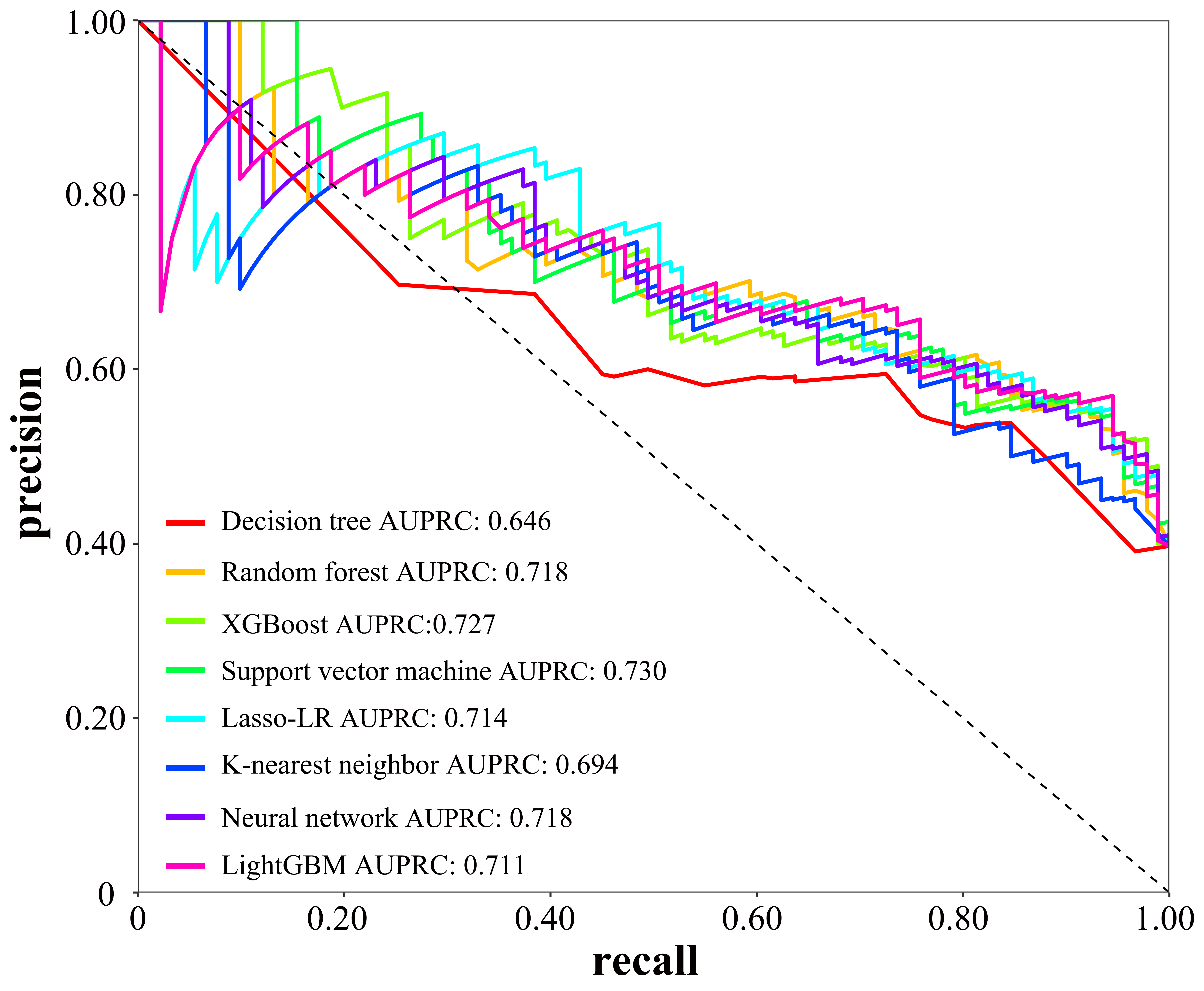

Supplement: Supplementary Figure 2 — AUPRCs of eight machine learning models in the temporal external validation cohort.AUPRC, area under the precision-recall curve; XGBoost, extreme gradient boosting; Lasso-LR, lasso-logistic regression; LightGBM, light gradient boosting machine. [file Image_2.tif]

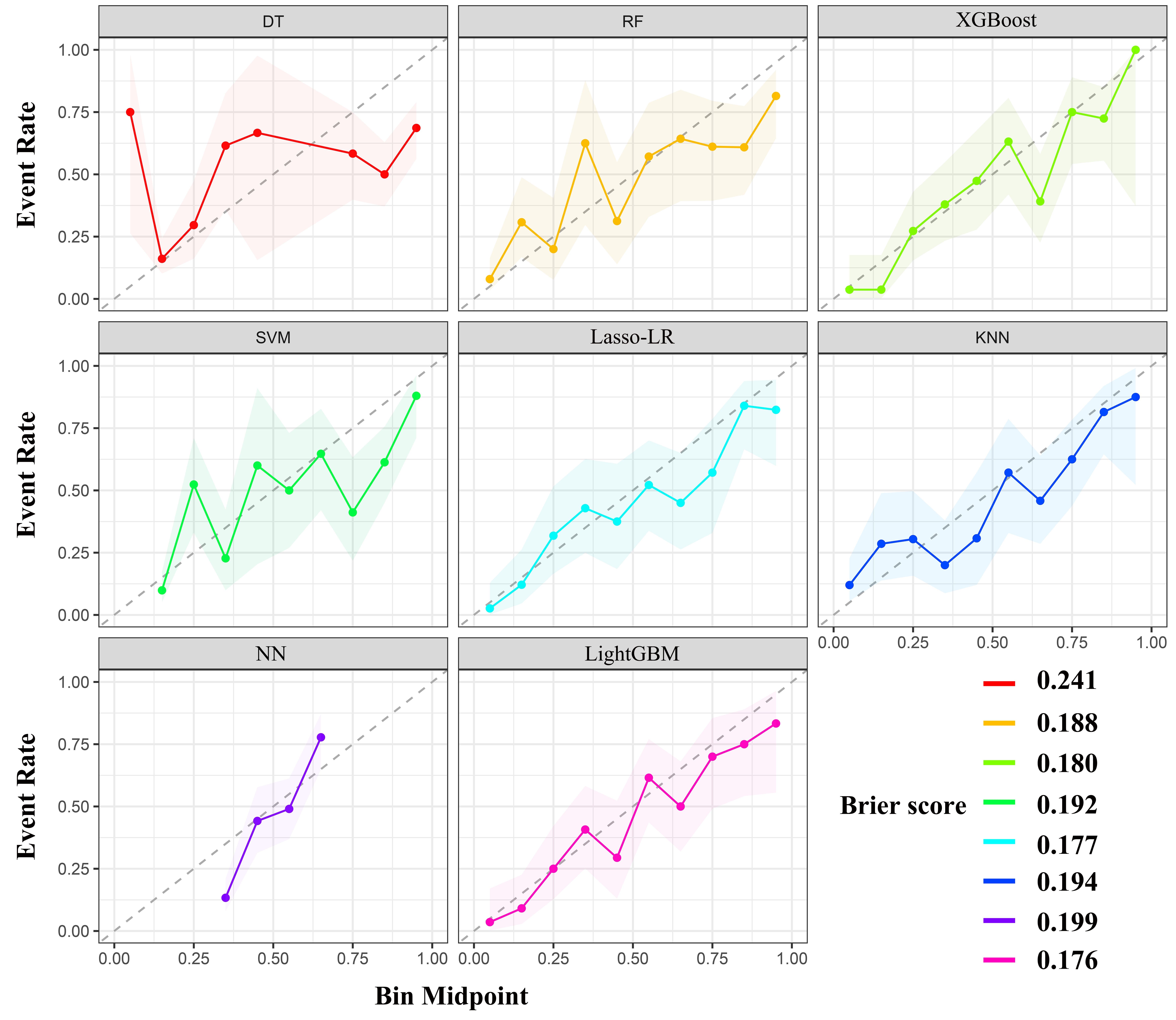

Supplement: Supplementary Figure 3 — Calibration curves of eight machine learning models in the temporal external validation cohort.DT, decision tree; RF, random forest; XGBoost, extreme gradient boosting; SVM, support vector machine; Lasso-LR, least absolute shrinkage and selection operator-logistic regression; KNN, k-nearest neighbor; NN, neural network; LightGBM, light gradient boosting machine. [file Image_3.tif]

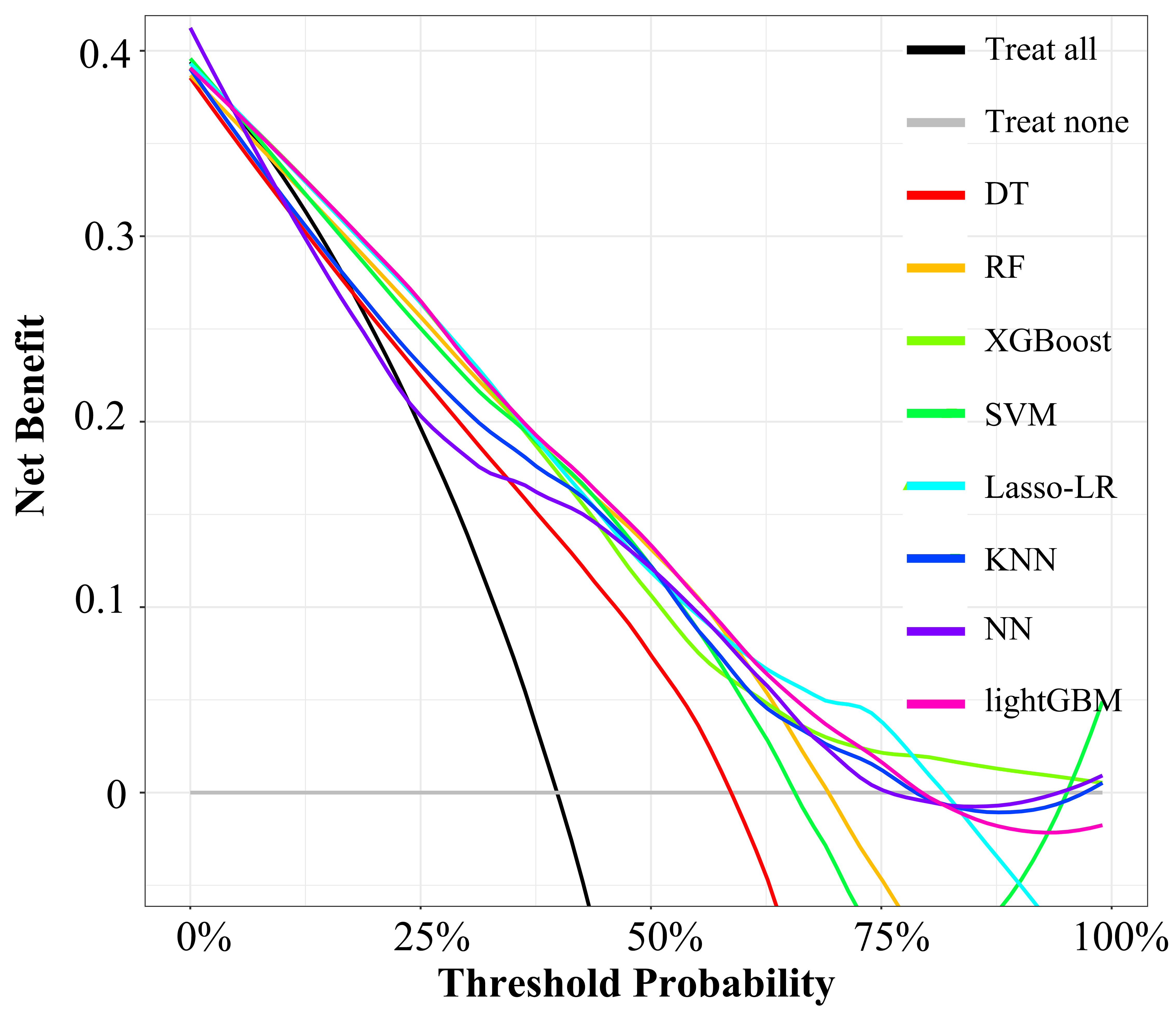

Supplement: Supplementary Figure 4 — Decision curve analysis of eight machine learning models in the temporal external validation cohort.DT, decision tree; RF, random forest; XGBoost, extreme gradient boosting; SVM, support vector machine; Lasso-LR, least absolute shrinkage and selection operator-logistic regression; KNN, k-nearest neighbor; NN, neural network; LightGBM, light gradient boosting machine. [file Image_4.tif]

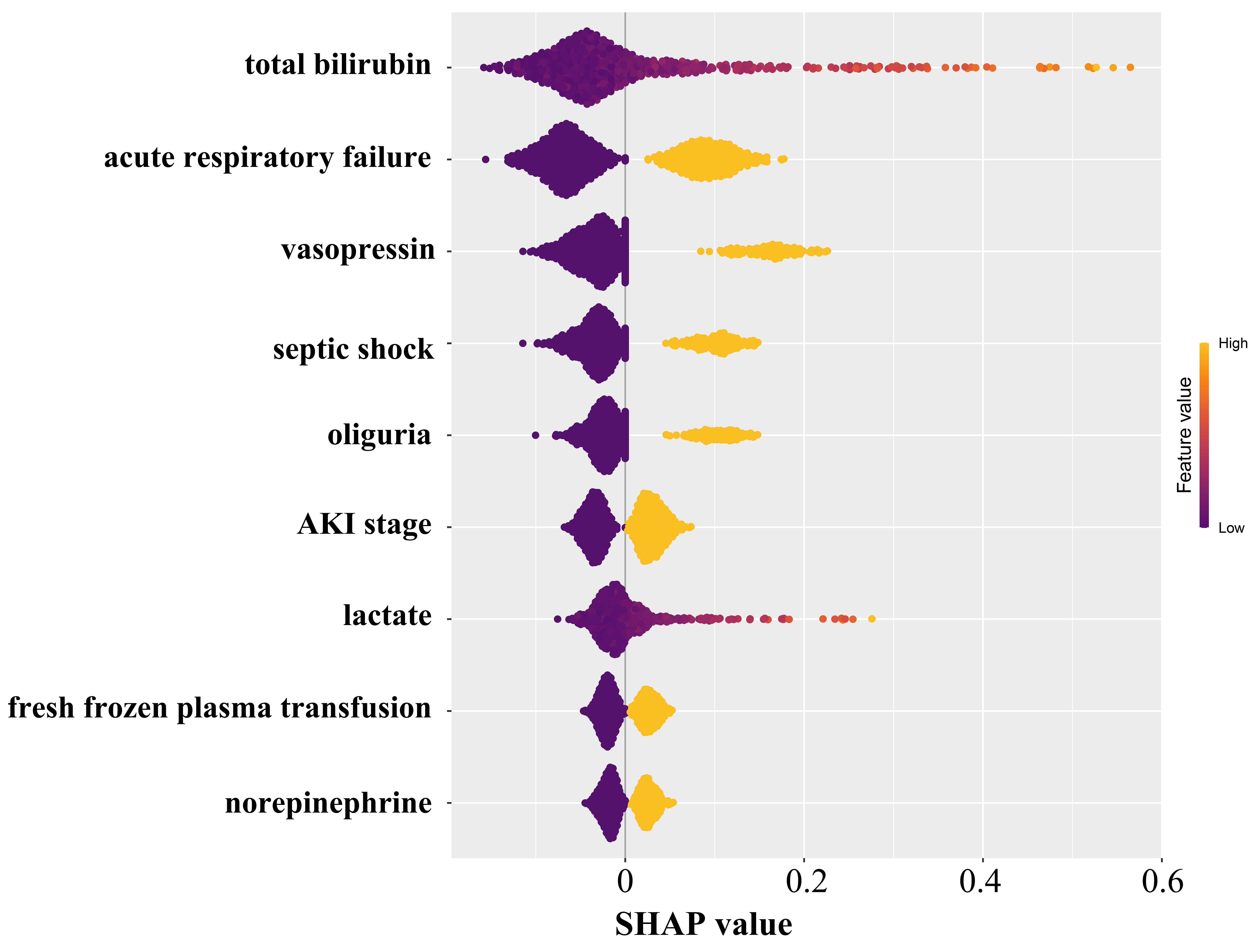

Supplement: Supplementary Figure 5 — Beeswarm plot of feature importance ranked by SHAP values in the Lasso-LR model.SHAP, SHapley Additive exPlanations; Lasso-LR, lasso-logistic regression; AKI, acute kidney injury. [file Image_5.tif]

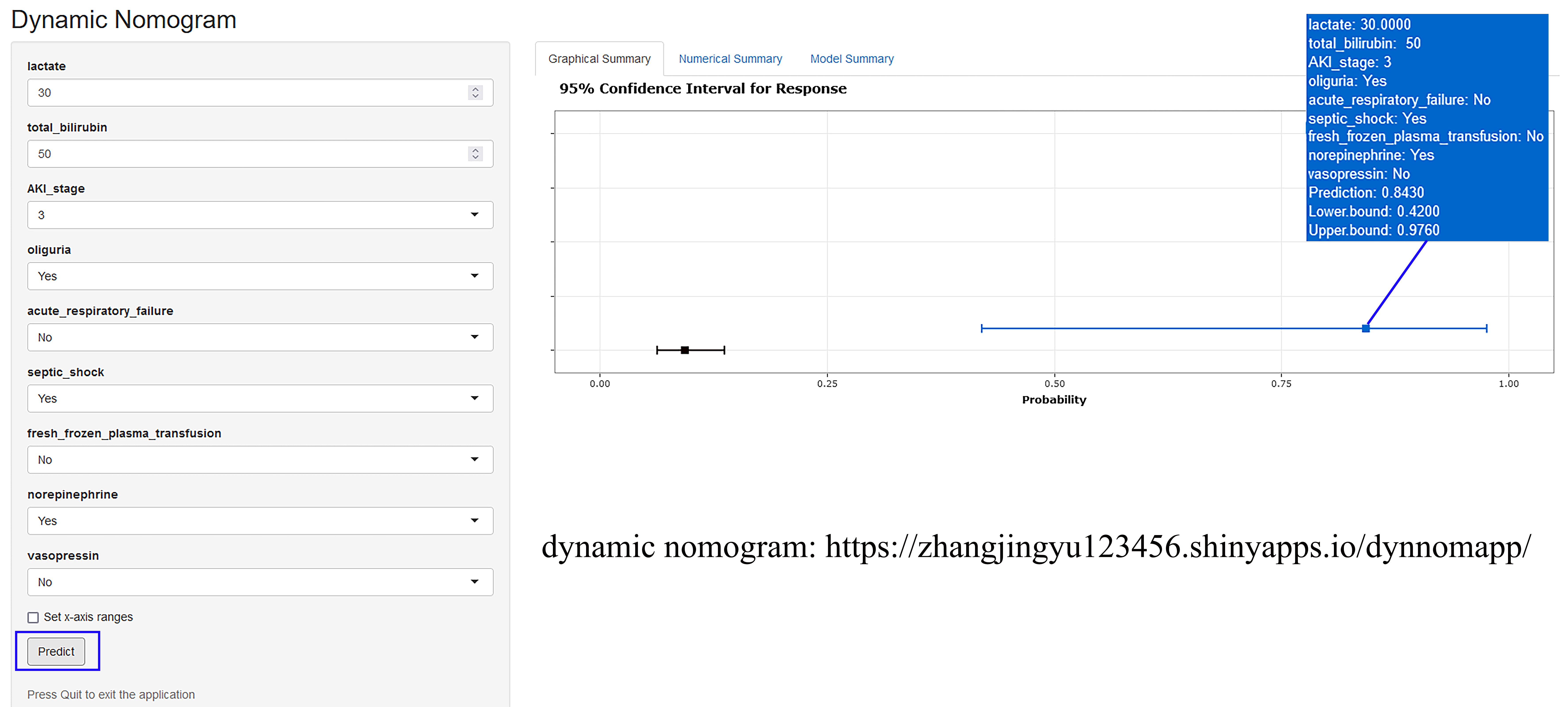

Supplement: Supplementary Figure 6 — Example case using the web-based dynamic nomogram. [file Image_6.tif]
